# SHEsisPlus, a toolset for genetic studies on polyploid species

**DOI:** 10.1038/srep24095

**Published:** 2016-04-06

**Authors:** Jiawei Shen, Zhiqiang Li, Jianhua Chen, Zhijian Song, Zhaowei Zhou, Yongyong Shi

**Affiliations:** 1 Bio-X Institutes, Key Laboratory for the Genetics of Developmental and Neuropsychiatric Disorders (Ministry of Education) and the Collaborative Innovation Center for Brain Science, Shanghai Jiao Tong University, Shanghai 200030, P.R. China; 2 School of Bio-medical Engineering, Shanghai Jiao Tong University, Shanghai 200230, P.R. China; 3 Institute of Social Cognitive and Behavioral Sciences, Shanghai Jiao Tong University, Shanghai 200240, P.R. China; 4 Shandong Provincial Key Laboratory of Metabolic Disease, the Affiliated Hospital of Qingdao University, 16 Jiangsu Road, Qingdao 266003, China; 5 Institute of Clinical Research, the Affiliated Hospital of Qingdao University, 16 Jiangsu Road, Qingdao 266003, China; 6 Shanghai Changning Mental Health Center, Shanghai 200042, P.R. China; 7 Department of Psychiatry, the First Teaching Hospital of Xinjiang Medical University, Urumqi 830054, P.R. China

## Abstract

Currently, algorithms and softwares for genetic analysis of diploid organisms with bi-allelic markers are well-established, while those for polyploids are limited. Here, we present SHEsisPlus, the online algorithm toolset for both dichotomous and quantitative trait genetic analysis on polyploid species (compatible with haploids and diploids, too). SHEsisPlus is also optimized for handling multiple-allele datasets. It’s free, open source and also designed to perform a range of analyses, including haplotype inference, linkage disequilibrium analysis, epistasis detection, Hardy-Weinberg equilibrium and single locus association tests. Meanwhile, we developed an accurate and efficient haplotype inference algorithm for polyploids and proposed an entropy-based algorithm to detect epistasis in the context of quantitative traits. A study of both simulated and real datasets showed that our haplotype inference algorithm was much faster and more accurate than existing ones. Our epistasis detection algorithm was the first try to apply information theory to characterizing the gene interactions in quantitative trait datasets. Results showed that its statistical power was significantly higher than conventional approaches. SHEsisPlus is freely available on the web at http://shesisplus.bio-x.cn/. Source code is freely available for download at https://github.com/celaoforever/SHEsisPlus.

During the last decade, SHEsis^1^ has become one of the most widely used tools for genetic association studies for diploids. However, polyploidy is common in plants. Association studies have been proven to be powerful approaches for identifying genes underlying complex diseases in human being^2^. Now the same strategy is being exploited in many plant species, along with the dramatic reduction in costs of genomic technologies. E.g. Huang *et al*. conducted a genome-wide association study (GWAS) in rice landraces and successfully identified loci associated with 14 agronomic traits^3^. Their work highlights the potential power of applying human genetic strategies to the investigation of genetic architecture of plants.

Here, we present SHEsisPlus (http://shesisplus.bio-x.cn/), an updated version of SHEsis^1^, to be an online platform for dichotomous and quantitative trait association analysis on polyploid datasets (also compatible with haploids and diploids). It’s free, open source and also designed to perform a range of analyses, including haplotype inference, linkage disequilibrium analysis, epistasis detection, Hardy-Weinberg equilibrium and single locus association tests.

Currently, there are two existing categories of computational methods for determining haplotypes: haplotype assembly and haplotype phasing. Haplotype assembly builds the haplotypes for a single individual from a set of sequence reads, while haplotype phasing attempts to infer the haplotypes using the shared haplotype information within the sample, given the genotypes of individuals from a population. Recently, two algorithms have been proposed for haplotype assembly: haptree^4^ and hapcompass^5^. However, not much progress has been made recently in developing the haplotype phasing algorithms for polyploids. Most of these algorithms are proposed several years ago. For example, SATlotyper^6^ uses the boolean satisfiability problem to formulate haplotype inference by pure parsimony. But it is not memory-efficient and its accuracy needs improving. PolyHap^7^ employs a hidden Markov model (HMM) and a sampling algorithm to infer haplotypes jointly. However, we found in simulation study that it is not compatible for multi-allelic markers and its accuracy decreases sharply for tetraploids.

Notably, epistasis also makes a substantial contribution to variation in complex traits such as disease susceptibility^8^. Although genome-wide studies have discovered a lot of variants associated with common diseases and traits, these variants typically appear to explain only a minority of the heritability. This “missing heritability” might be accounted for partly by epistasis^8^. Studies have also shown that epistatic effect might be present even when single-locus effects are minimal^9^. Methods for identifying interactive SNPs in case/control designs have been studied extensively^10^,^11^,^12^,^13^,^14^,^15^ while there have been few attempts to develop methods that systematically identify epistasis in quantitative traits^13^,^16^. The most conventional method to characterize epistasis for quantitative traits is linear regression. However, we found in our simulation study that, in the absence of main effects, the power of linear regression drops a lot. Entropy-based methods have been used for epistasis analysis for case/control studies and proves to be a promising direction because of its high power and capacity to detect pure epistasis^17^. Here we implemented this entropy-based algorithm in SHEsisPlus and extended it to the context of quantitative traits.

In this study, we implemented a user-friendly online platform for association analysis on polyploid species, developed an accurate and efficient haplotype phasing algorithm for polyploids and proposed an entropy-based algorithm to detect epistasis in the context of quantitative traits. Our haplotype phasing method uses the generalized greedy expectation maximization algorithm, which is an update of our previously proposed partition-ligation-combination-subdivision expectation maximization algorithm (PLCSEM)^18^. It is more efficient and more accurate than existing algorithms. And our epistasis detection algorithm is the first try to apply information theory to characterizing the gene interactions in quantitative trait datasets. A study of both simulated and real datasets showed that our algorithms significantly outperformed existing ones.

## Methods

### Haplotype inference

Our method is an update and generalization of our previously proposed partition-ligation- combination- subdivision expectation maximization algorithm (PLCSEM)^18^. First, loci are grouped into separate SNP blocks and loci within the same block are phased together. Then the phases are rebuilt hierarchically. This reduces the number of SNPs phased at one time and substantially improves speed. This also makes it easier for parallel computing. To deal with multi-allelic loci, we adopt the greedy expectation maximization (EM) algorithm to cut off the explosive increase in the number of possible haplotypes. Loci are phased one at a time by the EM algorithm and the phased loci are treated as a single multi-allelic locus. This strategy greatly reduces the computational complexity and makes it possible to phase the haplotypes of polyploid datasets within a very short time. The steps can be described as: (1) Split the loci into SNP blocks. (2) Within each block: a. Fetch the first 2 loci from the block. b. Infer the haplotypes of the 2 loci using the EM algorithm described below. c. Treat the 2 phased loci as a single multi-allelic locus. d. Fetch the next loci. Loop from a. to d. until all loci within the same SNP blocks are phased. (3) Rebuild the phase hierarchically. Treat the phased loci within each SNP block as a single multi-allelic locus and phase them just like how loci are phased within each block. (4) Loop until all the SNP are phased.

SNP blocks can be defined in multiple ways. The simplest way is to use a fixed partition size. Another way is to divide according to LD blocks. In our simulation study, we observed little difference when we tried different partitioning strategy. It is common knowledge that EM algorithm is likely to be trapped in a local optimum. To deal with this, different initial frequencies are assigned to the possible haplotypes. Loci are phased for multiple times using different initial value and the final solution is the one that is of the max likelihood. As SNPs are divided into blocks, this method ensures that optimum solution is more likely to be obtained within each block and thus, make it more likely to achieve the global optimum when all the SNPs are phased.

The procedure of EM algorithm^19^ for a p-ploidy datasets can be described as the following. First, the initial frequencies of each possible haplotypes are assigned. Here we assume that all possible haplotypes are equally likely:


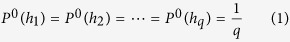


In the expectation step at the *g*^*th*^ iteration, the posterior probabilities of genotype assignments are calculated as:


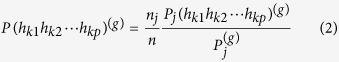


where *P*_*j*_ is the probability of the *j*^*th*^ phenotype, given by the sum of the probabilities of each of the possible genotypes, and 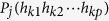 is the probabilities of genotype 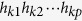 in phenotype *j*. In the maximization step, the next set of estimates of haplotype probabilities are obtained by summing posterior probabilities over instances of each distinct haplotypes:


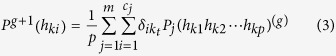


where 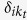 is an indicator variable equal to the number of times haplotype *k*_*t*_ is present in genotype *i*.

The performance of this algorithm was assessed using a real dataset from tetraploid potato genotypes, which was obtained from^6^, and a simulated dataset which was generated by randomly combining human male X-chromosomes from the HapMap project (See results).

### Epistasis detection

Interactions between SNPs are quantified by interaction information, which is the amount information bound up in a set of SNPs and is not present in any subset of these SNPs. Let’s first introduce some basic concepts in information theory.

### Entropy

Let’s assume an attribute, A. Shannon’s entropy^20^ is the measure of unpredictability of an attribute:


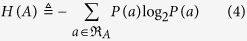


where *P*(*A*) is the distribution of attribute A. By definition, 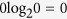.*H*(*A*) is the amount of uncertainty about A, as estimated from its probability distribution.

### Quantify attribute interactions

2-way interaction between 2 attributes can be quantified with mutual information:


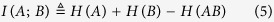


3-way interaction is measured by the intersection of all three attributes, or interaction information:


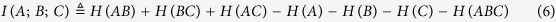


Then k-way interaction information can be generalized as:


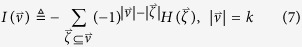


### Quantify SNP interactions

According to the definition of entropy, we can define the entropy of a bi-allelic SNP as:


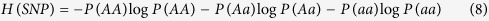


And the entropy of two bi-allelic SNPs can be defined as:


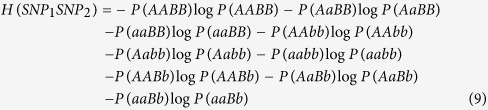


Then we can easily obtain the 2-way interaction between SNP_1_ and SNP_2_ by:


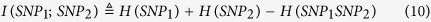


Generally, the k-way interaction of SNP set 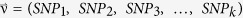 can be calculated by:


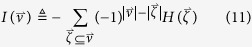


**Figure 1:**
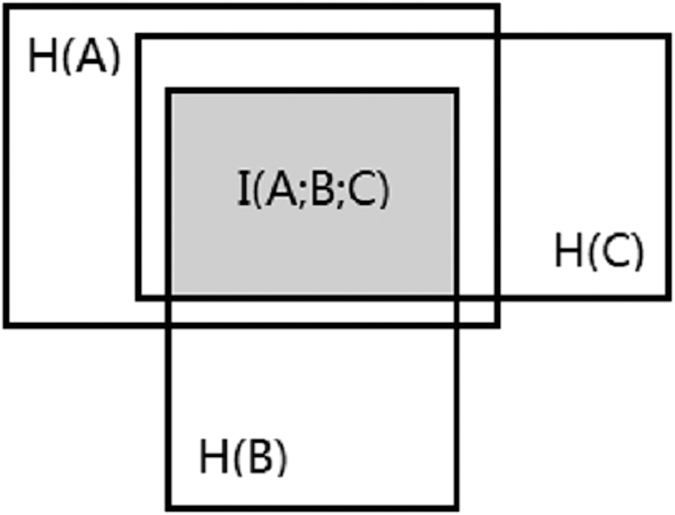
Illustration of 3-way interaction information. The intersection of H(A), H(B) and H(C) is the interaction information.

Figure 1 showed the illustration of 3-way interaction information.

### SNP interactions in quantitative trait datasets

Our algorithm for epistasis analysis is the first try to apply information theory to characterizing the gene interactions in quantitative trait datasets. For quantitative traits, individuals with some particular genotype combinations might have elevated trait values. So we aim to find out that if loci from individuals with higher trait values are more or less likely to interact than from those with lower trait values. To do this, we first classify individuals into high trait value group and low trait value group. We adopt Otsu’s method^21^ from computer vision and use it to find the optimum threshold separating the two groups so that their inter-class variance is maximal. In computer vision, Otsu’s method operates on the histogram of an image and automatically performs clustering-based image thresholding to reduce a gray level image to a binary image. In our case, the same procedure is performed on the histogram of the trait values to find the optimal threshold that best divides the samples. The inter-class variance is defined as:


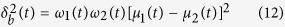


where,


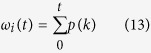



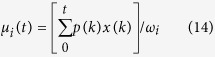



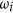
 are the probabilities of the two classes separated by a threshold *t* and *μ*_*i*_ are the class means. 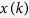 is the value at the center of the k-th histogram bin. P is the histogram of trait values.

The desired threshold corresponds to the maximum 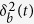.

After obtaining the threshold *t*, we can classify the individuals into two groups. Individuals with trait values higher than *t* are classified into the high trait value group while lower than *t* are classified into the low trait value group. Then interaction information is calculated in the groups respectively.

To get the k-way interaction of SNP set 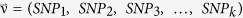, we first obtain:


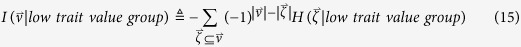



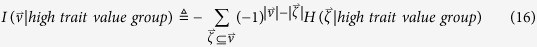


The difference in interaction information is:


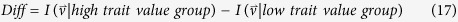


Then permutation test is performed to get the P value.

As this method does not test for the individual genotype combination; instead, it evaluates the overall difference across all the genotype combinations between the high trait value group and the low trait value group in order to increase the statistical power. Therefore, to find the risk genotype combinations, we generated the counts for each combination and used the standard chi square test. We also gave the odds ratio and p values for a certain genotype combination.

### SNP interactions in case/control datasets

For case/control studies, interactions information are calculated in cases and controls respectively.


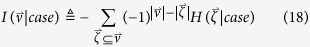



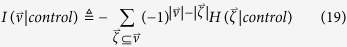


The difference in interaction information is:


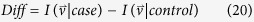


Then permutation test is performed to get the P value.

### Single locus association analysis

SHEsisPlus can adjust for covariates (age, sex, BMI, etc.) when performing single locus association analysis. For case/control design, if no covariates are provided, SHEsisPlus gives the results of Pearson’s Chi-square test and Fisher’s exact test for alleles and genotypes, else logistic regression will be used. The regression equation is:


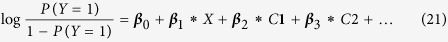


For quantitative traits, linear regression will be used instead. The regression equation is:


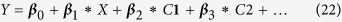


In the above equations, *Y* is disease status, *X* is genotype and 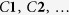, are covariates. The null hypothesis is 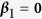.

### Hardy-Weinberg equilibrium test

The genotype frequencies in the Hardy-Weinberg equilibrium for a c-ploidy specie with n distinct alleles are given by individual terms in the multinomial expansion of:


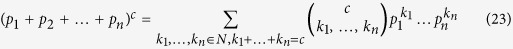


### Linkage disequilibrium analysis

For linkage disequilibrium analysis, normalized *D*’ and *r* are given, which can be calculated by:


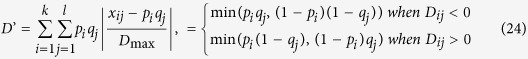



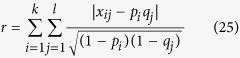


where 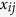 is the observed frequency of gamete *A*_*i*_*B*_*j*_, *p*_*i*_ and *q*_*j*_ are the frequencies of alleles *A*_*i*_ and *Bj* at locus *A* and *B*.

### Application of SHEsisPlus to serum uric acid level data for epistasis detection

#### Participants and Phenotypes

All the patients and controls were of Han Chinese origin and had long-term residence in the coastal areas of Shandong Province. A total of 622 unrelated cases were recruited from the gout clinic at the Affiliated Hospital of Qingdao University. All patients were diagnosed with primary gout by experienced physicians according to criteria established by the American College of Rheumatology. All 917 unrelated controls that had serum uric acid (SUA) values below 420 μmol/L, and never suffered from an acute attack of gouty arthritis were recruited. All participants with a family history of gout and severe illness, such as hepatitis or cancer, were excluded. This study was approved by the Ethics Committee of Affiliated Hospital of Qingdao University. All participants gave their written informed consent. The study was in accordance with the principles of the current version of the Declaration of Helsinki.

Phenotype details including age, height and weight were collected in a questionnaire at the time of admission and body mass index (BMI) was calculated from the calculation formula weight (kg)/height (m)^2^. All the samples were males. Systolic blood pressure (mmHg) and diastolic blood pressure (mmHg) were measured and recorded by physicians on our gout clinic. Related biochemical indicators including blood glucose, triglycerides, total cholesterol, urea nitrogen, creatinine and uric acid in the plasma were measured using an automated multichannel chemistry analyzer (Model 200; Toshiba, Tokyo, Japan).

Informed consent was obtained from all subjects. All experiments were performed in accordance with relevant guidelines and regulations which were approved by Shanghai Jiaotong University.

### SNP Selection and Genetic Analyses

To investigate whether the known serum uric acid level associated SNPs might interact with each other, we selected eight SNPs (rs12129861 at Chr1, rs780094 at Chr2, rs734553 at Chr4, rs742132 at Chr6, rs1183201 at Chr6, rs12356193 at Chr10, rs17300741 at Chr11, rs505802 at Chr11)^22^,^23^,^24^,^25^ , which were determined by a large-scale meta-analysis for SUA values (shown in Table 1). Genomic DNA was extracted from peripheral leukocytes according to the manufacturer’s protocols (Lifefeng Biotech Co., Ltd, Shanghai, China). Extracted DNA was confirmed and quantified with a NanoDrop 1000 Spectrophotometer (Thermo Scientific, USA). For the genotyping of these SNPs, PCR amplification was performed using the Gene Amp PCR System 9600 (Applied Biosystems, Foster City, CA, USA). 3% agarose gel electrophoresis was performed to separate the PCR products. Finally, DNA genotyping was performed using PRISM 3730 instruments (Applied Biosystems, Foster City, CA, USA). The primer sequences were designed using Primer 3 online Version 0.4.0 and obtained from Hanyu Biotech Co., Ltd, Shanghai, China.

### Statistical analysis

We performed linear regression to see if age and BMI might influence uric acid variability. The regression model is:


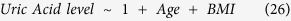


We found that BMI was significantly related to uric acid level (p = 1.49 × 10^−16^) while the correlation between age and uric acid level was weak (p = 0.48). Thus we adjusted uric acid level with respect to BMI. Epistasis were then evaluated by SHEsisPlus.

## Results

### Haplotype phasing in tetraploid potato genotypes

We applied SHEsisPlus to a real dataset from locus BA213c14t7 of *Solanum tuberosum*^6^. Locus BA213c14t7 corresponds to the sequenced T7 end of the BAC(bacterial artificial chromosome) clone BA213c14 and is located on potato chromosome V between the markers GP21 and GP179 near R1 gene for resistance to late blight^26^. This intergenic sequence region is characterized by high sequence variability. The dataset consisted of 19 heterozygous tetraploid individuals and 12 bi-allelic SNPs with known haplotypic phase obtained from laboratory. Although this resulted in 2^12^ possible haplotypes, SHEsisPlus reported that only 12 haplotypes existed in the current dataset, 9 of which were confirmed by the experimental phased results.

### Haplotype phasing in simulated dataset

Due to the limited availability of phased SNP data from polyploid species, we evaluated the performance of SHEsisPlus against large datasets by randomly combining human male X-chromosomes from the HapMap project to simulate diploid, triploid and tetraploid populations. We randomly chose a region that contains 30 heterozygous SNPs within the 6.4 Mb non pseudo-autosomal region of the X-chromosome (34, 135, 863 to 40, 527, 829 bp)^7^ and generated 10 datasets for each population. We used correct haplotype percentage (CHP) as a metric to compare the accuracy of different methods. CHP was defined as the percentage of ambiguous individuals whose haplotype estimates were completely correct^27^. This was a strict metric and was applicable when the number of involving SNPs was not too large. Table 2 showed that SHEsisPlus outperformed the existing methods on both accuracy and efficiency. For tetraploids, the accuracy of PolyHap decreased sharply to 78.91% while that of SHEsisPlus remained 98.14%. SATlotyper failed to calculate triploids and tetraploids because the scale of our simulated datasets was not computationally feasible for it.

To see if SHEsisPlus performed well when more loci were involved, we simulated larger datasets containing 100 SNPs and 1000 samples for diploids, triploids and tetraploids. We were unable to try the large datasets with polyHap and SATlotyper because of the computational burden. For large datasets, CHP was not an appropriate metric because as the length of the considered region increased, all methods would find it harder to correctly infer the entire haplotypes^27^. Instead, we used similarity index 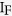, defined as the proportion of haplotype frequencies in common between estimated and true frequencies^19^:


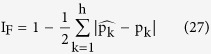


This would give more weight to common haplotypes. As SHEsisPlus was designed for association study, which focused on the difference in frequencies between cases and controls, we thought this metric would be more appropriate to fit this scenario. The 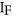 were 95.2%, 91.8%, and 87.9% for diploids, triploids and tetraploids, respectively.

### Power of epistasis detection algorithm in quantitative trait datasets

**Figure 2:**
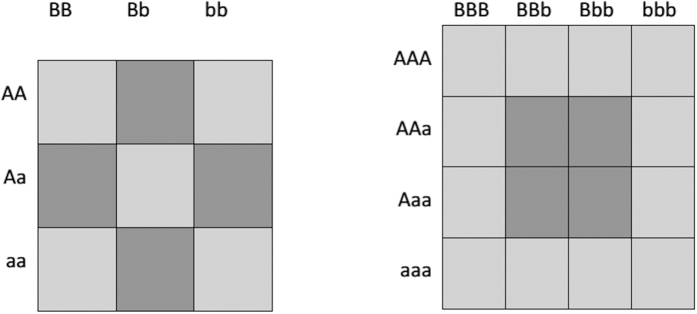
Epistasis models used for simulation study. (Left) Penetrance table for two-locus, bi-allelic epistasis in diploids (Right) Penetrance table for two-locus, bi-allelic epistasis in triploids.

As the power of this entropy-based algorithm for detecting epistasis in case/control data has been well-evaluated^17^, here we focus on its ability to characterize epistasis in the context of quantitative trait. We simulated datasets from 2-locus, bi-allelic, purely epistatic models. The penetrance tables were shown in Fig. 2. For diploids, the 4 double homozygotes and the double heterozygote genotypes have a mean trait value of 0 and the other genotypes containing exactly one heterozygote have an elevated mean trait value. For triploids, the double heterozygote genotypes have an elevated mean trait value while the others have a mean trait value of 0. The trait values are all generated from a normal distribution with a standard deviation of 1. Given random mating in an infinite population with equal allele frequencies at both loci, these two models are purely epistatic. For diploids, we compared the results with those of Plink (Plink is not applicable to polyploids).

The model that Plink uses to detect epistasis is linear regression. The regression model is:


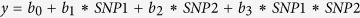


Test of interaction corresponds to testing whether the regression coefficient representing interaction terms (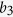) in the above formulation is zero. This can be done by the Plink option “−epistasis”.

**Figure 3:**
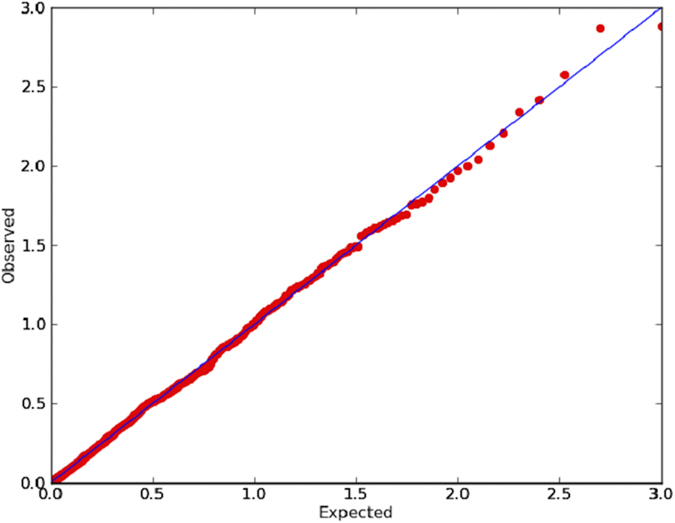
QQ plot of SHEsisPlus for 2-way epistasis detection in diploids in the context of quantitative trait. It approximately lied on the line y = x, indicating that the results were unbiased.

We found SHEsisPlus performed much better in identifying contributing epistatic loci than Plink^13^ (Tables 3, 4, 5, 6). Figure 3 is the QQ-plot of SHEsisPlus when calculating random data. We could see that it approximately lied on the line y = x, indicating that the results were unbiased.

### Application of SHEsisPlus to serum uric acid level data for epistasis detection

**Figure 4:**
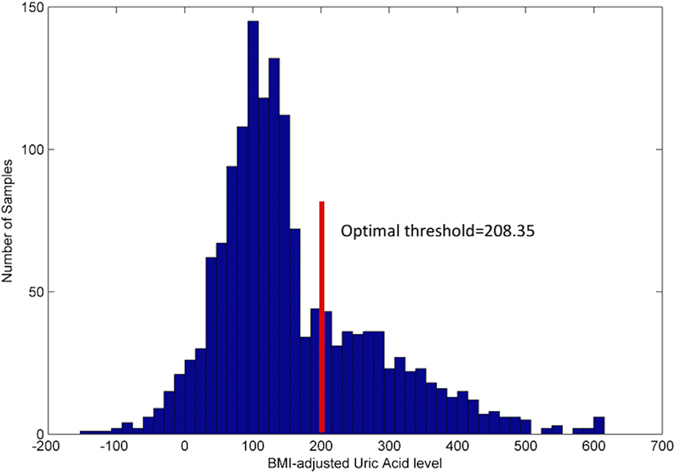
Distribution of the BMI-adjusted uric acid level. The optimal threshold to divide the samples is marked red.

We used SHEsisPlus to assess the 2-way to 8-way interactions between 8 loci (*PDZK1* rs12129861, *GCKR* rs780094, *SLC2A9* rs734553, *LRRC16A* rs742132, *SLC17A1* rs1183201, *SLC16A9* rs12356193, *SLC22A11* rs17300741, *SLC22A12* rs505802). The distribution of the BMI-adjusted uric acid level was shown in Fig. 4. The optimal threshold to divide the samples determined by our method was marked red. We could see that this threshold was approximately located in the valley of the two peaks. The results were listed in Table 7. Significant interactions after FDR correction were shown in bold. The most significant interaction was between rs12129861, rs742132, rs1183201 and rs12356193. In single locus analysis, only rs1183201 (p = 6.33 × 10^−4^, p = 0.001 after FDR correction) and rs12129861 (p = 0.022, p = 0.045 after FDR correction) showed significant association. Although rs12356193 and rs742132 didn’t show significant association with serum uric acid level, they, together with rs1183201 and rs12129861, exhibited strong interaction (p = 2.09 × 10^−6^, p = 5.16 × 10^−4^ after FDR correction).

## Discussion

In this paper, we developed a user-friendly online toolset for association analysis on polyploidy datasets with multi-allelic markers. We applied our method to both real and simulated datasets. Results showed that our haplotype phasing algorithm was much faster and more accurate than existing ones, especially for species with higher ploidy. The greedy expectation maximization algorithm is more efficient than the traditional EM algorithm because it significantly cuts off the explosive increase in the number of possible haplotypes for a certain genotype. However, it is common knowledge that EM algorithm is likely to be trapped at local maxima and consequently fails to reach global maxima. We deal with this problem by starting at different initial values and select the one with the highest likelihood as the final solution. Moreover, different partitioning strategies can be used to check if different results are obtained.

Our epistasis detection algorithm tries to apply information theory to characterizing gene interactions in quantitative trait datasets. Results showed that its power is much higher than linear regression, especially when the marginal effect is weak. For polypoids, as the number of genotype combinations is increased and the cells in the contingency tables become sparse, more samples are needed to achieve a relatively higher power. This is even more problematic for detecting high order epistasis. To solve this problem, we chose to divide the samples into two groups (the high trait value and low trait value groups). If the sample size within each group is small, the power will be limited. Therefore, it seems that the best strategy is to divide into as few groups as possible. We used the Otsu’s method to determine the threshold for sample division because it accounted for the pattern of the distribution. For the serum uric acid level dataset we used for epistasis analysis, the threshold determined by this method was approximately located in the valley of the two peaks, which was a reasonable threshold to classify the samples into high and low trait value groups. We found in our simulation study that Otsu’s method was robust because there was no extreme division generated by this method (e.g. too many samples in one group and too few in the other).

However, there are limitations of this method. It is not applicable to genome-wide datasets. It is designed for small datasets and one of its key features is that it can calculate the multi-way interaction. Multi-way interaction analysis is only applicable to small datasets because of the extremely high computational complex. However, in our previous work, we have proposed a GPU-based software (called SHEsisEpi^28^) for calculating genome-wide gene interaction, which can calculate the pair-wise gene interaction at a genome-wide scale. It can be downloaded from our website if needed. Download link: http://analysis.bio-x.cn/SHEsisMain.htm.

When applying SHEsisPlus to analyzing epistatic in serum uric acid level data, we found a novel interaction between rs12129861, rs742132, rs1183201 and rs12356193. rs12129861 is located in gene *PDZK1*, which encodes a scaffolding protein involved in assembly of a transporter complex in the apical membrane. rs1183201 is within gene *SLC17A1*, which encodes sodium phosphate transport protein 1. This protein mediates sodium and inorganic phosphate co-transport. Sodium-dependent transporter 1 has also been identified as a urate transport protein. All these 4 SNPs were reported by other researchers as serum uric acid level associated loci. But how they interact and confer risk to high serum uric acid level remains further study.

## Additional Information

**How to cite this article**: Shen, J. *et al*. SHEsisPlus, a toolset for genetic studies on polyploid species. *Sci. Rep*. **6**, 24095; doi: 10.1038/srep24095 (2016).

## Figures and Tables

**Table 1:** Summary of eight SNPs used in analysis.

SNP	Position^a^	Gene name and function	Allele^b^	Populations	Allele frequency*
					Allele CEU CHB
rs12129861	1q21.1	PDZK1, 5′Intergenic	G/A	European	A 0.460 0.170
rs780094	2p23.3	GCKR, Intron16	G/A	European	A 0.394 0.566
rs734553	4p10.1	SLC2A9, Intron7	A/C	European	C 0.261 0.004
rs742132	6p22.2	LRRC16A, Intron34	T/C	European, Japanese	C 0.301 0.244
rs1183201	6p22.2	SLC17A1, Intron 3	T/A	European	A—
rs12356193	10q21.2	SLC16A9, Intron 5	A/G	European	G 0.186 0.141
rs17300741	11q13.1	SLC22A11, Intron4	A/G	European	G 0.531 0.073
rs505802	11q13.1	SLC22A12, 5′Intergenic	G/A	European	A 0.726 0.256

^a^On human genome build 18.

^b^In NCBI.

^*^Collected from HapMap Data Phase III/Rel#3. CEU: Utah residents with Northern and Western European ancestry from the CEPH collection, CHB: Han Chinese in Beijing, China.

**Table 2:** Accuracy and running time of SHEsisPlus for haplotype inference.

Algorithm/Ploidy	2	3	4
SHEsisPlus	99.63% (6.317 s)	98.74% (15.862 s)	98.14% (51.109 s)
PolyHap	99.15% (12.25 m)	98.21% (3.075 h)	78.91% (43.95 h)
SATlotyper	90.46% (19.80 m)	–	–

**Table 3:** Power of SHEsisPlus for epistasis detection in diploids.

Samples	sd*	alpha = 0.05 SHEsisPlus/Plink	alpha = 0.01 SHEsisPlus/Plink
2000	0.25	0.494/0.041	0.331/0.006
2000	0.5	0.779/0.047	0.693/0.005
2000	0.75	0.870/0.054	0.832/0.004
2000	1	0.923/0.056	0.902/0.004
2000	1.5	0.948/0.065	0.938/0.008
2000	2	0.966/0.066	0.950/0.009
2000	2.5	0.971/0.064	0.968/0.009

*Number of standard deviation apart between two groups.

**Table 4:** Power of SHEsisPlus for epistasis detection in triploids.

Samples	sd*	alpha = 0.05	alpha = 0.01
2000	0.25	0.099	0.024
2000	0.5	0.24	0.115
2000	0.75	0.418	0.287
2000	1	0.602	0.472
2000	1.5	0.824	0.762
2000	2	0.901	0.863
2000	2.5	0.903	0.877

*Number of standard deviation apart between two groups.

**Table 5:** False positive rate of SHEsisPlus for epistasis detection in diploids.

Samples	alpha = 0.05 SHEsisPlus/Plink	alpha = 0.01 SHEsisPlus/Plink
500	0.055/0.048	0.008/0.013
1000	0.047/0.053	0.009/0.007
2000	0.051/0.051	0.008/0.015
3000	0.033/0.060	0.008/0.015
5000	0.052/0.058	0.012/0.011

**Table 6:** False positive rate of SHEsisPlus for epistasis detection in triploids.

Samples	alpha = 0.05	alpha = 0.01
500	0.042	0.006
1000	0.043	0.008
2000	0.045	0.012
3000	0.044	0.007
5000	0.054	0.009

**Table 7:** SHEsisPlus results on the uric acid level data.

SNP set	P value	FDR
rs742132,rs12356193	0.005	0.176
rs1183201,rs12356193	0.001	**0.044**
rs12129861,rs742132,rs505802	6.03e–04	**0.03**
rs12129861,rs1183201,rs12356193	6.13e–04	**0.03**
rs12129861,rs12356193,rs505802	0.01	0.288
rs734553,rs742132,rs1183201	0.028	0.638
rs12129861,rs780094,rs742132,rs12356193	6.26e–04	**0.03**
rs12129861,rs742132,rs1183201,rs12356193	2.09e–06	**5.16e–04**
rs12129861,rs742132,rs12356193,rs505802	0.008	0.261
rs12129861,rs742132,rs17300741,rs505802	0.041	0.86
rs12129861,rs780094,rs742132,rs1183201,rs12356193	7.72e–05	**0.009**
rs12129861,rs780094,rs742132,rs1183201,rs12356193,rs17300741	0.017	0.42
